# Exploring the influences on men’s engagement with weight loss services: a qualitative study

**DOI:** 10.1186/s12889-020-8252-5

**Published:** 2020-02-25

**Authors:** Megan Elliott, Fiona Gillison, Julie Barnett

**Affiliations:** 1 Faculty of Life Sciences and Education, University of South Wales, Lower Glyntaff Campus, Pontypridd, CF37 1DL UK; 2 Department for Health, University of Bath, Bath, BA2 7AY UK; 3 Department of Psychology, University of Bath, Bath, BA2 7AY UK

**Keywords:** Obesity, Overweight, Weight loss, Men, Male, Engagement

## Abstract

**Background:**

Engagement of men with commercial and UK National Health Service (NHS) weight loss services is low, and few studies report on why this may be. However, evidence shows that men who do participate in weight loss programmes tend to lose as much, or more weight than women. The present study aimed to explore men’s experiences and expectations of mainstream weight loss services in the UK, following referral from a medical professional, particular in relation to barriers and motivators.

**Methods:**

Semi-structured interviews were conducted with 18 men with a BMI over 25 kg/m^2^ including those who had, and had not, attended group-based or one-to-one weight loss services. Interviews were analysed using thematic analysis.

**Results:**

Two themes were identified; *'Fear as a motivation for change'* (1) and *'Attitudes towards existing weight loss services'* (2). Within theme two, two subthemes were identified; *‘Female dominated services’* and *‘Incompatibility of existing services for men’.* The findings suggest that fear, as a result of a medical diagnosis or referral is a mechanism for motivating men to engage with weight loss services. This was often augmented by awareness of other people’s experiences of poor health due to their weight. The gender imbalance and attitudes towards existing weight loss services deterred men from engaging with or continuously attending sessions. This imbalance resulted in feelings of self-consciousness, shame and a perceived stigma for men using weight loss services. These experiences highlighted the importance of providing services which align with men’s preferences to promote engagement.

**Conclusions:**

A medical diagnosis or referral serves as a strong motivator for men to engage with weight loss services by invoking fear of negative consequences of not losing weight. Men perceived weight loss services to be feminised spaces, in which they felt self-conscious and out of place. As a result, men were deterred from engaging and considered their options were limited. Implications for service design and commissioning are discussed. Involving men in research, service design and evaluation is key to improving their engagement and weight loss.

## Background

The prevalence of overweight in the United Kingdom (UK) adult population is a major public health concern, as more than 63% of men and 56% of women have a body mass index (BMI) over 25 kg/m^2^, classifying them as overweight [1–4]. The prevalence of overweight is particularly high for adults over 45-years (> 70%) [1–5]. Public Health England [6] estimated that in 2014–15, obesity and overweight cost NHS England £6.1 billion and society £27 billion overall [1]. Increased BMI is associated with a number of health problems, including type 2 diabetes, hypertension and cancer [1, 5, 6]. Each additional 5 kg/m^2^ in BMI is associated with a 30% increase in overall mortality, a 40% increase in vascular mortality and a 60–120% increase in diabetic, renal and hepatic mortality [7].

A modest weight loss of 5–10% of initial body weight has been associated with numerous clinical benefits. These include a decline in progression to diabetes by up to 58% over 4 years [8], as well as reduced risk of cardiovascular disease and lowered blood pressure [9–11]. Aside from the clinical benefits, weight loss has been associated with feeling healthier, younger, more positive and getting better quality sleep [12]. As evidence suggests that weight loss programmes can produce a weight reduction that is accompanied by clinical [9–11] and psychological [9] benefits, men who do not engage are not benefiting from these services. Greater prevalence of overweight in men, compared to women in the UK [1–4] and growing obesity rates globally [13] increase the risk of associated health issues in men, and demonstrate the potential value of enabling behaviour changes, such as improved diet or increased physical activity, to mitigate these risks. However, while knowledge of ways to lose weight may be high, this is often not successfully translated into behaviour change and help and supervision from a healthcare professional may be required to promote weight loss and maintenance [14, 15].

In most areas of the UK, support for individuals to lose weight is provided through referral to group or one-to-one weight loss programmes such as Counterweight and Slimming on Referral. Counterweight [16, 17] is an NHS commissioned community-based lifestyle programme, consisting of six fortnightly sessions focusing on varying aspects of dieting or behaviour and weight monitoring, as well as maintenance and review sessions. Slimming on Referral [18, 19] is a 12-week complimentary referral to commercial weight loss providers, e.g. Weight Watchers and Slimming World. These programmes also promote diet change, provide exercise plans and involve group sessions providing social support. Both services can be accessed through self-referral directly to the programme, or through general practice (GP) referral to weight loss specialists. Both Counterweight [16, 17] and Slimming on Referral [18, 19] have been found to be successful in supporting patients to lose weight over the course of the programme. However, these and other similar programmes have low rates of engaging men. Only 23 and 24.9% of Counterweight participants were men when delivered in primary care [16] and community pharmacies [20] respectively, and engagement within commercial slimming programmes are even lower than for NHS-commissioned programmes like Counterweight. On average, 86% of participants in Slimming on Referral services are women [18], with men representing only 11–18% of participants across Weight Watchers, Slimming World and Rosemary Conley Diet and Fitness Clubs. In part this may be reflected by lower rates of referral for men, for example Lavin et al. [19] reported that only 11% of referrals to commercial slimming programmes from GPs were men.

Men are also under-represented in weight loss research. A systematic review of 244 randomised controlled trials (RCT) of weight loss programmes (*N* = 95,207) found that only 27% of participants were men [21]. This figure was slightly greater for trials that recruited from populations with comorbid diseases, such as type 2 diabetes or hypertension. Of the studies included in the systematic review, only 5% exclusively recruited men, as opposed to 32% recruiting women-only samples. A more recent systematic review [22] supported this, finding that of RCTs for weight loss interventions open to men and women, only 36% of participants were men. Low engagement of men in weight loss programmes, and the under-representation of men in the related literature is problematic for researchers and services as findings from weight loss studies using all or predominantly women may not be generalisable to men. Gender differences may mean that programmes that have been proven to reduce weight in women may be less effective in men.

Despite low engagement, the evidence generally shows that men who do participate in weight loss programmes tend to lose as much, or more weight than women. A systematic review of studies directly comparing programme outcomes in men and women found that, out of 21 studies, 11 reported significant gender differences for weight loss, with ten reporting that men lost more weight than women [23]. Similarly, a study of 1.3 million self-referrals to Slimming World [24] reported greater body weight percentage reductions for men (5.7%), compared to women (4.3%), however only 5% of participants were men. Furthermore, more men were classified as higher attenders and men lost weight significantly faster than women. A systematic review [22] found no significant differences in amount of weight loss between men and women, but found that men and women responded differently to different weight loss programmes. For example, men lost more weight with intensive low-fat diets and structured exercise programmes than women. In order to inform strategies to increase engagement with existing programmes or to inform the design of new ones it is therefore crucial to understand and address reasons for low engagement of men in existing effective weight loss programmes.

Few studies have considered factors that might impact upon the engagement of men in weight loss services [7]. Possible explanations that have been highlighted include; men not viewing weight as an issue [25], greater misclassification of overweight among men [26], men perceiving dieting as feminine [12] and viewing weight loss facilities as feminised spaces [27], construal of diet as women-centred and ‘unmasculine’ in the media [28] and men associating dieting with unpalatable foods, small portions and restrictions [12]. Meanwhile, humour, male-oriented banter and inclusion of physical activity [22] have been found to facilitate attendance of men. However, no studies have primarily focused on generic reasons for low engagement rates and have rather tended to gather men’s views and opinions of specific weight loss programmes [12].

### The present study

In the present study we sought to answer the research question; *“What influences engagement of men in weight loss services?”* We aimed to report on the barriers that men report independently of any specific weight loss service, by recruiting participants who have engaged with a range of different weight loss services, both commercial and those provided through the UK NHS, and individuals who have declined referrals to weight loss services.

## Methods

### Design

The present study employed a qualitative, cross-sectional design using semi-structured interviews with overweight and obese men. Qualitative interviews were selected as the most appropriate method of data collection, as they allow participants to freely express their thoughts and opinions [29] and discuss topics that had not previously been considered [30]. An initial sample of 15–20 participants was sought, in accordance with Braun & Clarke’s [31] recommendations for qualitative interview studies, with potential to supplement this with further recruitment if data saturation was not reached. The study was conducted in accordance with the British Psychological Society recommendations for research. Ethical approval was received from an Institutional ethics committee (18–165).

### Participants

#### Eligibility criteria

The target participants were men who had been referred by a medical professional to a weight loss service as they had a BMI over 25 kg/m^2^ (i.e., clinical definition of overweight [1]) with or without comorbidities.

#### Sampling and recruitment

Existing clients or new referrals to a Healthy Lifestyle Service for weight loss support in south west England were asked by practitioners if they may be interested in participating in a research study. With their consent for contact, a researcher subsequently invited them to participate by telephone. Thirty-five individuals were contacted by phone, 12 were not reachable and two declined to participate. Prospective participants (*n* = 21) were provided with an information sheet prior to the interview by post or e-mail and written consent was obtained when the participant arrived for the interview. Three participants did not attend their scheduled interview, without giving a reason.

To facilitate interpretation of participant comments relative to their individual context, they were asked about their previous experience of weight loss services and from this classified as; ‘completers’ if they had completed at least 12-weeks of a single programme; ‘current participators’ if they had attended one or more sessions, but not yet completed a programme; ‘non-engagers’ if they had declined or had not yet taken up their referral to a programme and; ‘repeaters’ if they had engaged in two or more programmes in the past.

### Data collection

A semi-structured interview schedule was constructed in accordance with guidelines for improving trustworthiness and rigour [32]. The five phases; identifying prerequisites for semi-structured interviews (1), reviewing and using existing literature (2), creating the interview schedule (3), pilot testing the schedule (4) and reporting the full schedule (5; See Additional file 1) were carefully followed. The interview schedule aimed to explore; history and effects of weight gain, motivations for help seeking and engagement, experiences of weight loss services, reasons for non-engagement, barriers, challenges and preferences.

Interviews were conducted in a private room within two hospitals (*n* = 14) and a university (*n* = 4) in June and July 2018 by the first author (Female, Health Psychology MSc). Interviews were audio-recorded and transcribed verbatim by the interviewer, and lasted an average of 38 min (shortest = 12; longest = 61). Transcriptions were checked against audio recordings by the first author for accuracy. Participants were debriefed and given a £10 voucher as token of gratitude for their contribution.

### Analysis

Data analysis was conducted using thematic analysis. This approach was informed by a critical realist stance [33]. Critical realism proposes that a real and knowable world exists, but it can only be accessed through subjective and socially-located knowledge [34]. As such, the truth can only be known through the social world, culture and history [31] and qualitative methods, like interviews, can be used to access this subjective knowledge.

To ensure rigorous data analysis, Braun and Clarke’s [35] recommendations for applying a structured approach to analysis, using a six-phase approach: (1) Familiarisation with data was facilitated through listening to the audio recordings and reading the transcriptions multiple times. (2) Initial codes were generated by the first author through an inductive process of identifying meaning units, and developing descriptive codes (*n* = 171) sticking closely to words use by participants themselves, using NVivo 11 Pro. (3) The first author then worked to cluster similar codes together and identify where duplication and clarity were needed. Each code was written on card and manually grouped into clusters, which were refined into draft theme, and then (4) shared and reviewed alongside coded transcripts and indicative quotes with both co-authors, before (5) discussing and agreeing the definitions and names of themes. (6) The report was then produced, and refinements and clarifications of themes finalised in order to communicate the results more clearly. The analysis was iterative, moving bi-directionally through the phases to ensure a thorough analysis process [35], see Table 1.

**Table 1:** Codes, clusters and themes generated through thematic analysis

Code	Cluster	Subtheme	Theme
Motivation; Family weight issues, experiences of others	Motivation		Fear as a motivation for change
Medical problems; doctor instructions; involvement of medical staff; referral; reluctance to see GP; told what to do	Medical drivers		
Fear; consequences of weight; fear of ageing	Fear		
Trigger; weight creeping on; denial of weight problems in the past; kidding yourself	Denial until trigger		
Women’s club; female environment; program experiences; drop out; feeling self-conscious	Minority	Female dominated services	Attitudes towards existing weight loss services
Gender differences; opinions about men; stigma; being manly; social pressures; feeling self-conscious; competition	Male identity		
Social pressures; social life; stigma; female influence (wife/partner); sense of belonging	Lack peer support		
Gender-sensitised programmes; patronising, embarrassing; program experiences; female environment; opinions about groups	Format of typical services	Incompatibility of existing services for men	
Commercial weight loss preconceptions; misperceptions; preconceptions; strict diets; calorie counting	Preconceptions		

## Results

Eighteen participants (average age 59 years) were recruited (see Table 2). All participants had a BMI over 25 kg/m^2^ (overweight) and the majority (*n* = 15) had a BMI over 30 kg/m^2^, classifying them as obese. There were three ‘completers’, five ‘current participators’, three ‘non-engagers’ and seven ‘repeaters’. Of those who had engaged in a weight loss programme (*n* = 15), nine engaged with an NHS delivered programme (Counterweight), four engaged with a commercial weight loss programme (Weight Watchers or Slimming World) and two had engaged in both types. Engagement in Counterweight was in either group sessions (*n* = 4), on a one-to-one basis (*n* = 6) or both (*n* = 1). Commercial weight loss programmes were only delivered as group sessions.

**Table 2:** Participant characteristics. CW-Counterweight, SW-Slimming World, WW-Weight Watchers, G-Group, I-Individual

ID	Age Range	BMI	Weight classification	Ethnicity	Work status	Education level	Programme status	Programme(s) engagement
1	65–74	31.5	Obese	White British	Retired	No qualifications	Completer	CW (G)
2	55–64	43.9	Obese	White British	Not working	Diploma	Current participator	CW (I)
3	35–44	28.4	Overweight	Arabic	Not working	Graduate	Current participator	CW (I)
4	75+	38	Obese	White British	Retired	Postgraduate	Repeater	CW (I), SW (G)
5	65–74	31.1	Obese	White British	Part-time	Graduate	Completer	CW (G)
6	55–64	30.6	Obese	White British	Full-time	Graduate	Repeater	CW (I)
7	35–44	46.2	Obese	White British	Full-time	Diploma	Non-engager	None
8	65–74	34	Obese	White British	Full-time	School leaver	Repeater	CW (I), SW (G)
9	45–54	30.5	Obese	White British	Part-time	School leaver	Non-engager	None
10	55–64	33.3	Obese	White British	Full-time	Diploma	Current participator	CW (I)
11	65–74	35.5	Obese	White British	Retired	School leaver	Repeater	CW (I, G)
12	65–74	27.4	Overweight	White British	Retired	Postgraduate	Completer	CW (G)
13	55–64	36.6	Obese	White British	Full-time	School leaver	Repeater	SW (G)
14	45–54	33.2	Obese	White British	Full-time	Diploma	Non-engager	None
15	65–74	32.2	Obese	White British	Part-time	No qualifications	Current participator	SW (G)
16	35–44	37.4	Obese	White British	Full-time	School leaver	Repeater	CW, SW, WW (G)
17	75+	25.9	Overweight	White British	Retired	No qualifications	Current participator	SW (G)
18	55–64	31	Obese	White British	Full-time	Graduate	Repeater	WW (G)

Two themes were identified; *'Fear as a motivation for change'* (Theme 1) and *'Attitudes towards existing weight loss services'* (Theme 2). Within theme 2, two subthemes were identified; *‘Female dominated services’* and *‘Incompatibility of existing services for* men’. Illustrative quotes are provided to support the themes, identified by participant number, programme status and type of programme (NHS Service/Commercial).

### Theme 1: Fear as a motivation for change

For many participants, a medical diagnosis invoked feelings of fear and worry, with one participant saying that the diagnosis *“put a bit of the frighteners”* [*P1, Completer, NHS Service*] on him and subsequently led him to engage in a weight loss programme.

In these cases, a medical diagnosis created an external trigger, causing a *“shock to the system”* [*P5, Completer, NHS Service*], which the men interviewed felt was needed to drive their intentions to lose weight. Most commonly, this was a diagnosis of a weight-related health condition, such as; high blood pressure, diabetes, hip and knee troubles or heart problems. This diagnosis, coupled with instructions and a referral from a healthcare professional, motivated men to take up their referral to a weight loss service:

*“I had my blood pressure taken for a routine, sort of annual MOT, and whereas in the past it’s always been fine, this last time it was high, so I asked my GP what I should do about the blood pressure, and she said, one thing you can do is to lose weight” [P12, Completer, NHS Service]*

In addition to a diagnosis, being made aware of alternative, more severe measures that may be necessary to reduce weight in the future scared participants and served as a strong motivator:

*“My weight had gone up to 160kg … and my GP hinted at well you don’t want to carry on putting on weight because you might have to have a gastric band and I said no, we're not going to go there” [P2, Current participator, NHS Service]*

For another participant, the motivating factor was losing weight to access surgery for knee pain:

*“I have these problems with my knee and they said I’ve got to lose 10% of my body weight before they can think about operating, so that’s why I decided to do it” [P1, Completer, NHS Service]*

Knowledge of the implications and irreversible damage caused by obesity also induced fear for one participant:

“*I understand the causes of diabetes and how close I am to it, and the fatty liver that I’ve got, and if I don’t do something about it now, the irreversible damage it’s causing” [P7, Non-engager]*

Participants confessed that without this *“kick-start”* they would not have changed their behaviour or sought out weight loss support:

*“I’ve been in there [the doctor’s] about various things … lethargic and all the rest of it, and yeah, … it [the diagnosis] was a kick up the backside that I needed” [P18, Repeater, Commercial]*

*“I wouldn’t have ever thought about going there [the weight loss group] and doing it without being referred, I’d never even heard of it” [P1, Completer, NHS Service]*

Fears that not losing weight would continue to impair participants’ mobility and prevent them from living their lives as normal were also effective in motivating behaviour change:

*“The excess weight was having a negative, very painful effect … I was having a lot of problems just walking around” [P3, Current participator, NHS Service]*

In addition to receiving a medical diagnosis of a weight-related condition, men were also motivated by other people’s experiences of poor health that had resulted from being overweight. The consequences of obesity in family members or friends often augmented participants’ personal motivation, and helped them to understand the severity of their condition:

*“I lost my sister-in-law … for her whole life she had a massive weight problem, and I put it down, you know, at the end it cost her life” [P8, Repeater, Commercial & NHS Service]*

For this participant, seeing the consequences of long-term weight issues on family members highlighted the association between obesity and ill health. However, even where this link was evident, for one participant with a family history of obesity, this association may lead to perceptions of inevitability and a lack of control:

*“Both my brothers were obese as well and they all have other medical issues as well, along with myself … my dad had a heart condition, diabetes, liver condition, problems with his kidneys and again that was all mostly down to obesity” [P7, Non-engager]*

Both ‘engagers’ and ‘non-engagers’ discussed their fears and awareness of the potential negative consequences of obesity. This suggests that whilst these fears may be effective in motivating engagement for some, other factors many mean that they are not always sufficient or effective in promoting behaviour change.

### Theme 2: Attitudes towards existing weight loss services

Despite the power of medical triggers and fears discussed in Theme 1, men were still often reluctant to engage in weight loss services or reporting dropping out of services, particularly commercialised programmes. They reported a number of barriers that have been previously identified in the literature, including time, practical and financial constraints, convenience of unhealthy foods and the challenge of integrating weight loss with a busy lifestyle. However, a particularly strong theme among this sample was the men’s attitudes, expectations, feelings and experiences about the nature of existing weight loss services. This theme will be explored within the context of two sub-themes; ‘*Female-dominated services’* and *‘Incompatibility of existing services for men’.*

#### Subtheme 1: Female-dominated services

Men found themselves in the minority when attending weight loss services, particularly commercial services and this was perceived negatively:

*“Only me and two other guys and about 40 women [at Weight Watchers], which is a bit off-putting” [P4, Repeater, Commercial & NHS Service]*

*“It’s usually all women [at Slimming World], there are a couple of men there … it’s a bit patronising” [P15, Current participator, Commercial]*

Being in the minority provoked feelings of self-consciousness and embarrassment in men:

*“I suppose I almost would be self-conscious about going into a weight loss thing when there were men and women there, you know I’d feel a bit silly … So I think the more male one may be, is a good idea” [P10, Current participator, NHS Service]*

One participant explained that the sense of shame was particularly related to discussing the sensitive topic of weight loss with women, rather than any discomfort related to being in the minority in general:

*“The fact that it’s mainly female … don’t get you wrong I only had female employees, I was very used to dealing with women. But I didn’t feel comfortable in a group like that” [P5, Completer, NHS Service]*

Another participant discussed how it takes a lot of confidence to attend these services, and, although he was able to attend, he believed that the majority of men would feel embarrassed or ashamed about attending, and acknowledged his own feelings on the matter:

*“I know I’m OK because I’m, broad shoulders, but I know from speaking to other people that it’s really tough walking into a female environment, like a weight loss environment, because you feel like a failure … you shouldn’t be there … like a women’s club, that’s what it’s like … it doesn’t feel good really” [P13, Repeater, Commercial]*

Attending a female-dominated weight loss service threatened their identity as men; it was not perceived as a ‘manly’ thing to do:

*“I think it’s just the stigma that’s attached to it for weight loss for men, they think it’s more of a woman’s thing to lose weight and worry about their weight” [P7, Non-engager]*

These feelings were perpetuated by the fact that men rarely discussed the topic of weight loss with friends. This may be due to a male *“bravado”* [*P18, Repeater, Commercial*] - not wanting to show that they were self-conscious or concerned about their weight. Whilst one participant believed that discussions about weight amongst men were becoming more normalised, he still believed that the stigma about obesity would prevail, making accessing peer support difficult:

“A male talking about a bloody diet, if you were to talk to me 20 years ago, I would’ve said f*ck off! But now when you go out, men do talk about weight and things like that … culturally it’s changed now … But I think there is a stigma still with being fat, I just don’t think that’s going to go away” [P14, Non-engager]

The lack of male peers in weight loss services, combined with a perceived stigma about engaging with services and dieting represents a strong barrier for men to seek support and engage with services that promote weight loss. 

#### Subtheme 2: Incompatibility of existing services for men

Men often discussed how the weight loss services that they had engaged with did not align with their preferences. This resulted in discomfort, and was particularly evident in the examples of participants not fully engaging in the services; i.e. only attending for the weight monitoring aspect and leaving before the discussion:

*“So lots of men like me, go and get weighed and get out. Usually the older men stay … but it’s, it doesn’t do it for me” [P13, Repeater, Commercial]*

*“No, I’m in and out, I can’t be doing with all this bloody talking and all this” [P17, Current participator, Commercial]*

Participants felt that the group discussion aspect of the session was tailored to the needs and preferences of women, with a large focus on discussing their weight loss and praising each other for their achievements, which was considered to be intrusive and overbearing:

*“All clap each other and that, that doesn’t work for me, because that’s quite embarrassing for me. They have like a slimmer of the week and all that stuff, that doesn’t work for me” [P13, Repeater, Commercial]*

*“Slimming World, I went once and went back the second time, and I just didn’t, it just wasn’t for me … I just found a bunch of people nattering, I don’t need that” [P8, Repeater, Commercial & NHS Service]*

Thus, men reported that the nature of these weight loss services either put them off starting services or deterred them from engaging fully or continuing. However, while these experiences cemented participant’s views that existing services are not always appropriate for them, participants’ views of men-only services in the present study were mixed.

Some participants felt that creating services exclusively for men would encourage engagement, as they may feel more comfortable and less self-conscious about sharing their feelings and experiences than in a mixed group:

*“Probably be easier for men to have just men … you obviously will share more with men than you will with women” [P4, Repeater, Commercial & NHS Service]*

Participants also highlighted the value of male-orientated ‘banter’ and being able to talk openly and honestly with other men:

*“More male conversations and things like that, so I don’t know, if there was a female within that, that would change the dynamic” [P14, Non-engager]*

However, others felt that being in a group, regardless of the gender distribution, was valuable because members were all able to offer advice and help each other. Some men even felt that a female influence was important for initiating conversations in group sessions:

*“Men don’t really … talk, you might not get anything out of it” [P16, Repeater, Commercial & NHS Service]*

One participant actually suggested that a male-only environment may be detrimental to overall success because it might fuel unhealthy competition and put unnecessary pressure on attendees:

*“Mixed groups are better, there’s … less pressure if you’re in a mixed group than if you put a bunch of fifty-something blokes together, they’d all want to out-do each other!” [P6, Repeater, NHS Service]*

In contrast, there were also participants who held a strong preference for individual, one-to-one sessions:

*“There’s lots of sort of things where you’re in front of everybody else, and they sort of clap when you’ve lost some weight. And I thought, no that’s not for me, which is why the one [one-to-one sessions] I’m doing now is quite good” [P10, Current participator, NHS Service]*

The variety of views highlights the differences in men’s preferences for weight loss services, suggesting the importance of offering a variety of service formats, which are more compatible with the preferences of men.

## Discussion

The themes in the present study offer a novel insight into what influences men’s engagement with weight loss services. Two themes were identified: ‘*Fear as a motivation for change’* and *‘Attitudes towards existing weight loss services'.*

Fears about further health deterioration, negative consequences of being overweight or the need for surgical interventions for weight were common amongst the men we interviewed. Medical diagnoses and referrals from medical professionals also appeared to invoke fear in these participants. Protection Motivation Theory (PMT) [36] posits that efficacy of a fear appeal in producing attitude change depends on three components; (1) the severity of a threatening event, (2) the perceived probability of occurrence, vulnerability, and (3) the perceived efficacy of a protective response. For those who perceived that attempts to lose weight could be successful in reducing their risk of complications, fear of the consequences of continuing to be overweight appeared to have a positive, motivating effect, resulting in engagement with a weight loss programme. However, PMT could also offer an explanation as to why this fear did not motivate engagement in all participants. For those who perceived the severity and vulnerability to be too much, and did not believe that weight loss attempts would be sufficient, the intensity of the fear may have reduced their perceptions of self-efficacy and inhibited their motivation to change their behaviour.

For the majority of participants, fear resulted from a medical consultation or medical consequences, which motivated them to engage in weight loss services. This is reflected in the literature; help seeking for weight loss [37] and higher uptake of weight loss services [21] is observed in men when a medical comorbidity exists, when a man is referred by their GP [7] or when a man perceives that his health is at risk [38]. Other qualitative research with men has also found that receiving information about their health risk status and weight motivated men to engage with a programme [39]. Ours and other research thus suggests an important role for GPs in increasing the number of men referred to weight loss treatment. While encouraging GPs to do so is consistent with current UK policy, for example Making Every Contact Count (MECC) [40], GPs are often reluctant to raise the issue of weight in consultations [41] because of the risk of damaging their patient relationship or discouraging patients from future engagement with healthcare services. Training GPs to raise the issue of weight in brief consultations in an empathic and non-stigmatising manner [39], by using techniques such as motivational interviewing [42, 43], may increase their confidence and referral rates. Despite this, men are also less likely to receive a referral as they are 32% less likely to attend the GP compared to women [44], suggesting the importance of engaging with men in alternative, non-medicalised settings, e.g. supermarkets or sports clubs.

Perceptions that weight loss services were female-dominated and designed for women represented a barrier for many of the men that we interviewed. This made engaging with services, particularly commercial services with a strong gender imbalance, especially challenging for men. Other qualitative studies of men in weight loss programmes also found that male attendees feel uncomfortable, embarrassed and ostracised for being a minority [45]. Men also felt that existing services were tailored to women, which they perceived to be inappropriate for meeting their needs. Whilst these perceptions were not necessarily powerful enough to stop participants from engaging altogether, they did impact upon their level of engagement, with some men only attending part of a session, leaving before group discussions, or dropping out after a couple of sessions. Satisfaction with services is a strong predictor of service attendance [46], and subsequently, attendance predicts better weight loss outcomes [16]. This suggests that low satisfaction with a service, due to these perceptions may hinder attendance and weight loss in men.

Some participants reported feeling self-conscious when attending groups in which men represented a small minority. They identified that these feelings were specific to the weight loss context, as opposed to any group setting with a predominance of women. They felt that worrying about weight was a ‘woman’s thing’, and that being seen to be concerned or talk about dieting challenged their self-identity and masculinity. Other studies have also reported on the incompatibility of the construct of masculinity with weight loss in men [﻿38﻿, 47]. For example, contemporary social norms about body size for men dictate that the ideal body size is big and strong [38] and taking part in ‘feminine’ activities, e.g. commercial weight loss groups, has been said to reduce ‘man points’ and masculinity [48]. Participants also commented that they would rarely discuss the topic of weight with their peers, resulting in a lack of social support for their weight loss attempts. Peer support or peer mentoring interventions may be effective in transforming social norms and men’s thoughts about weight loss [49].

A potential way to address these negative perceptions and barriers may be to deliver weight loss services that specifically target men, align with their preferences for humour, male-oriented banter and physical activity, and fit with their masculine constructs [7, 50]. The Football Fans in Training (FFIT) programme [51] is an example of a unique initiative that has been successful in doing so. The 12-week programme, hosted in a sporting venue, gives men a strong sense of affiliation in a context that confirms their male identity [7]. The programme has high retention rates (~ 90%), is effective in promoting and maintaining weight loss in men [50]. The FFIT model has since been successfully implemented and tested with men in other sport settings, including rugby [52] and hockey [53]. Transferability of the model to other sports may capture a wider audience, but still relies on strong identification to a local sports club, which may not appeal to all men. The ‘SHED-IT’ website-based weight loss programme has also been successful in appealing to men [54] and uses comical language and a non-intrusive, flexible approach to recruitment. Despite the successes of these programmes, the participants in the present study had mixed views regarding men-only programmes, with some suggesting they may become overly competitive or lack useful discussions that may be facilitated by women. Therefore, offering a range of services, delivered as mixed-group, men-only and one-to-one may be effective in increasing engagement, attendance, satisfaction and outcomes for a range of men.

### Limitations

The participants in the present study were not ethnically diverse, with 94% being White British, although this mirrored the population where the research was conducted [55]. Furthermore, the age range of participants in the present study was 36–78 years. Whilst UK obesity and overweight are most prevalent in the 55–64 years age group [1–4], obesity rates are also high in younger adults, who are less likely to engage with existing weight loss services [56]. Inclusion of younger adults in the sample would have broadened findings and offered insight into the experiences and barriers for this particular age group.

Additionally, the first theme identified the importance of a medical trigger for engaging with weight loss services. This may be confounded by the fact that all participants recruited into the study had already received a referral from a medical professional to a health improvement service for their weight. Seeking perspectives from overweight men who had independently enrolled onto a weight loss service, or who recognise they are overweight but have not sought support, may identify other important triggers and motivations for engagement.

No member checking or other triangulation was attempted as part of this research, which would have provided further confidence in the veracity of the themes and interpretations. We believe that data saturation was reached for participants who had engaged with at least one programme, however recruitment of non-engagers was low. Whilst interviews with engagers yielded some insight into barriers and reasons for non-engagement, additional interviews with non-engagers may have allowed for data saturation within this sub-group and given further insight into why some men do not engage with weight loss services.

Finally, the interviewer for this study was a healthy-weight young woman. Given the issue that some participants raised about feeling self-conscious in discussing their weight in female-dominated groups, this could have made overweight male participants feel self-conscious about discussing their weight and health, and less forth-coming during the interviews.

## Future recommendations

Further work would be useful to explore the preferences and experiences of understudied men, such as those from ethnic minorities, younger men, those living in remote/rural areas and gay, bisexual or transgender men [12]. Prospective studies and RCTs investigating efficacy of weight loss services for men may help identify underlying mechanisms, establish if they are different from women, on which the majority of the literature is based, and could contribute to a better understanding of how to prevent weight gain and reduce obesity in men. Future work would be valuable to explore whether attaining better gender balance within existing services would be sufficient to reduce perceptions that weight management services are a female domain, and consequently increase engagement, or whether men’s perceptions about weight contradicting their self-identity would still provide a challenge to engaging with services.

## Conclusions

The present study provides some insight into the barriers men face to engaging with and using mainstream weight loss services. The findings suggest that fear motivates men to engage, but negative perceptions about the female-dominated and incompatible nature of existing weight loss services, particularly commercial services, deter men from engaging. Understanding motivations and barriers to engaging is key to addressing them and reducing rising rates of obesity, overweight and associated weight-related health problems.

## Supplementary information

**Additional file 1.** Interview schedule.

## Data Availability

The datasets used and analysed during the current study are available from the corresponding author on reasonable request. The interview schedule is supplied in the Supplementary Materials.

## Abbreviations

BMI: Body Mass Index
CW: Counterweight
FFIT: Football Fans in Training
G: Group
GP: General practice, General practitioner
I: Individual
MECC: Making Every Contact Count
NHS: National Health Service
P: Participant
PMT: Protection Motivation Theory
RCT: Randomised controlled trial
SW: Slimming World
UK: United Kingdom
WW: Weight Watchers
