# Supplementary material for: Exploring the influences on men’s engagement with weight loss services: a qualitative study
Source: BMC Public Health. 2020 Feb 25;20:249. doi: 10.1186/s12889-020-8252-5 (PMC7041184; doi:10.1186/s12889-020-8252-5)
Supplement: Supplementary file 1 — Additional file 1. Interview schedule. [file 12889_2020_8252_MOESM1_ESM.docx]

**INTERVIEW SCHEDULE**

*Investigating the views of males regarding existing weight loss programs and the design of future weight loss programs*

Opening

First of all, I would like to thank you for taking the time to talk to me. As you know, I am interested in understanding your thoughts and opinions about weight loss programs that are offered by the Healthy lifestyle service, *particularly relating to your experiences as a man.*

I am going to record the interview so that I can listen to what you have said at a later date. However, if you want to stop the interview or the tape recorder at any time you can. All the information that you provide will be strictly confidential. Your name will not be mentioned in any reports arising from this study.

Section 1:

- Could you tell me about your weight history and how you think you gained weight?
  - Prompts: long-term struggle with weight, sudden change, trigger for weight gain
- Weight management program participation
  - Have you ever participated in a formal weight-loss program?
  - If yes…
    - Was this within the last: Five years, two years, one year, 6 months, 3 months, currently participating.
    - What program was it? Counterweight, Slimming World, Weight Watchers, other
    - Did you complete the full program?
    - Were you referred by your GP or did you self-refer?
    - *GO TO 2A*
  - If no…
    - *GO TO 2B*

2A. Experience (past/present) of weight loss programs (15 mins)

- So, you’ve said that you have tried *[INSERT PROGRAM NAME HERE]* in the past. Could you tell me about your experiences with this?
  - Prompts:
    - How was the program?
    - How did you find out about the program?
    - Did you have any prior knowledge/preconceptions/expectations about the program?
    - What was good about it?
    - What did you not like?
    - What would you have changed?
    - What was the outcome of the program for you?
    - How did it make you feel?
    - Did the program give you the results you wanted? Did you maintain the weight loss?
- What motivated you to lose weight?
- What made it easy for you to attend?
- Were there any barriers which put you off attending?

2B. Those who declined offers for weight loss programs (15 mins)

- Which program were you offered? Were you offered a range of programs?
- Why did you turn these down?
- What were the barriers that stopped you from participating?

3. Tier 1+ program reception

The Healthy Lifestyle Service is currently in the process of redesigning their weight management services. We are trying to create a new type of care that supports people in managing their own weight, using a very brief intervention, without regular input from healthcare professionals or group sessions.

This would include 3-5 15 minute sessions with a specialist where they would give you some initial information and also signposting you to other resources, like websites, Apps or printed booklets taking information from Public Health England One You, NHS Choices and Change 4 Life, and relying on you to monitor your own weight and diet yourself.

These include peer support through online forums, using behaviour change booklets and worksheets, or using a BMI tracker to monitor your weight changes.

- How does this sound to you?
- What kind of support would appeal to you?
- Do you think you would be able to do this?
- Can you think of any barriers to this?
- Have you got any other thoughts about this type of program?

4. Future program design

Now I would like to do an activity with you… I would like you to pretend that it is your job to design a weight loss program for men with a BMI over 25. Take a minute to think about the kind of program you would choose, how you would run the program and how you would get people to come along?

Be as creative as you want.

Tell me some of the features of the program…

Prompts:

- Mode of delivery
- Duration/frequency
- Group/individual
- Gender sensitised (men only?)
- Location – less threatening venues
  - Who would deliver it?
- Style of sessions – what would you do in it?
- Focus of sessions (e.g. calories, exercise)
- Incentives
- Social support/competitive
- Recruitment – how to recruit? Where from?
- Digital basis

Thank you for everything you’ve told me, it’s been really interesting for me. Finally, could I just ask you some demographic questions, as mentioned on the information sheet, these won’t be used in a way that could identify you.

- How old are you?
- What is your highest level of education?
- What is your work status at the moment? (full time/part time/shift work/retired/unemployed)
- And finally, your height and weight.
- Do you know your BMI?

Thank you very much for participating.

*Debrief and offer debrief form*.
